# Experimental study on the heat treatment reaction process of bentonite

**DOI:** 10.1038/s41598-024-67555-z

**Published:** 2024-07-19

**Authors:** Long Hai, Jiarui Wang

**Affiliations:** School of Mechanics and Engineering, Liaoning Technical University, Fuxin, 123000 Liaoning China

**Keywords:** Bentonite, Heat treatment, Temperature, Energy, Microstructure, Engineering, Materials science

## Abstract

This study focuses on enhancing the pozzolanic activity of bentonite through heat treatment to improve its compressive strength, while also considering its expansion properties for applications. Sodium bentonite was subjected to various temperatures and analyzed using thermogravimetric-differential scanning calorimetry (TG-DSC), X-ray diffraction (XRD), and scanning electron microscopy-energy dispersive spectroscopy (SEM–EDS). The results indicated that at 100 °C, adsorbed and interlayer water in montmorillonite was lost, and constitution water was eliminated at 700 °C. With further temperature increases, montmorillonite decomposes into an amorphous phase at 900 °C. At 1100 °C, the amorphous phase recrystallized into magnesium–aluminum silicate, which further decomposed into cristobalite. The study concludes that bentonite heat-treated at 800–900 °C can be effectively used as an additive in mining backfill materials to enhance compressive strength while maintaining its expansion properties.

## Introduction

**Figure 1:**
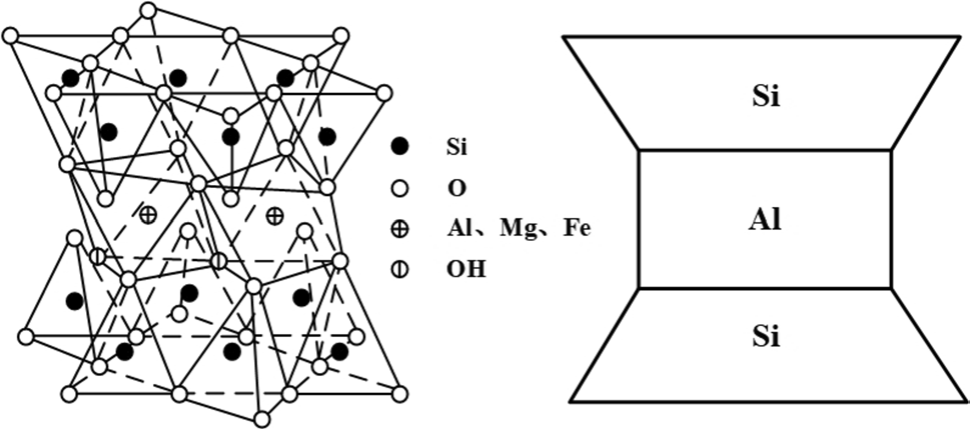
Crystal structure of montmorillonite.

Bentonite is primarily composed of montmorillonite. Montmorillonite is a hydrated layered aluminosilicate mineral belonging to the monoclinic crystal system, and its chemical formula can be represented as Al_2_O_3_•4SiO_2_•nH_2_O. It has a typical 2:1 layered structure, consisting of two silicon-oxygen tetrahedral layers sandwiching an aluminum-oxygen octahedral layer ^1^, as shown in Fig. 1.^2^. Due to the breakage of chemical bonds and the presence of charges at the edges, montmorillonite crystals exhibit strong electrostatic attraction, which allows them to pull polar water molecules between the layers, leading to expansion. The entry of water increases the interlayer spacing, which then permits even more water molecules to enter between the crystals. Ultimately, the cations adsorbed between the layers of the montmorillonite crystals diffuse onto the crystal surface, forming a double layer, which results in an excess of like charges on the crystal layers. This causes the crystal layers to repel each other due to similar charges, further accelerating the expansion of the interlayer spacing. Thus, montmorillonite determines the swelling and water absorption properties of bentonite^3,4^.

In addition, bentonite may also contain illite, quartz and other phases, and its main chemical composition is usually SiO_2_, Al_2_O_3_ and Fe_2_O_3_, which, in combination with cement, can consume free lime and form additional C-S–H gels^5^. Bentonite is a good additive for concrete materials because it can consume more silicate than other volcanic ash materials, especially kaolin^6^.

With the increasing demand for mineral resources, shallow mineral resources are becoming increasingly depleted, and deeper underground mineral resources are gradually being exploited^7,8^. A large amount of solid waste is generated during the mining of deep mineral resources, which affects the ecological environment in and around mining areas^9^. The filling mining method allows reasonable utilization of solid waste resources and ensures the stability of a quarry^10^, but there are still many technical engineering difficulties, including the inability for the backfill material to completely reach the roof^11^. Barati et al.^12^ mixed bentonite with tailings sand to investigate the effect of bentonite mixing on the compressive strength of tailings sand and demonstrated that bentonite combined with tailings sand can be used as a filling material. Zhang et al.^13^ studied the effect of different doses of bentonite on the performance of backfill materials and reported that the expansion rate increased with increasing bentonite dose, resulting in an increase in the rate at which the backfill material reached the roof. Yu et al.^14^ conducted uniaxial compression tests on 0%, 5%, 10% and 15% bentonite-doped specimens of a filled body and found that the uniaxial compressive strength and modulus of elasticity decreased and then increased with increasing bentonite dose, but the compressive strength of the specimens after increased bentonite doping was still lower than that of the undoped specimens. In addition, Zhao et al.^15^ investigated the mechanical properties of engineered cementitious composites doped with bentonite and found that the addition of bentonite decreased the specimen compressive strength. Ghanizadeh et al.^16^ prepared two groups of bentonite plastic concrete specimens, with each group consisting of 72 specimens, and similarly found that the addition of bentonite decreased the compressive strength of the specimens. Therefore, using natural bentonite as an additive to improve the backfill material, can expand the backfill material and effectively improve the rate of roof contact, but there is a problem with compressive strength reduction.

Thermal activation of bentonite through heat treatment can enhance its pozzolanic activity^17–19^, which in turn increases the compressive strength of the material and addresses the issue of reduced compressive strength. However, previous studies on using heat-treated bentonite as an additive for concrete have primarily focused on enhancing its pozzolanic activity through heat treatment to boost compressive strength, paying less attention to the effects of heat treatment on the expansion properties of bentonite. Xu^20^, through XRD analysis, discovered that the montmorillonite phase disappears when the heat treatment temperature reaches 600°C. To achieve higher pozzolanic activity in bentonite and improve the compressive strength of concrete, 800°C was selected as the optimal heat treatment temperature based on uniaxial compression test results. The disappearance of the montmorillonite phase indicates a loss of expansion properties in bentonite. For mining goaf backfill materials, it is essential not only to increase the compressive strength of the material but also to maintain its expansion properties to improve roof contact efficiency. Exploring a heat treatment temperature for bentonite that both enhances compressive strength and retains some expansion properties is crucial for the development of expansive backfill materials for mining applications.

To determine the optimal heat treatment temperature range, this study investigates the thermal reaction process of bentonite using TG-DSC, XRD, and SEM–EDS methods. This research findings aim to broaden the application scope of heat-treated bentonite in mine backfill materials and the concrete construction industry, providing experimental evidence for the use of bentonite in fields related to thermal activation and modification.

## Experiment

### Experimental material

**Figure 2:**
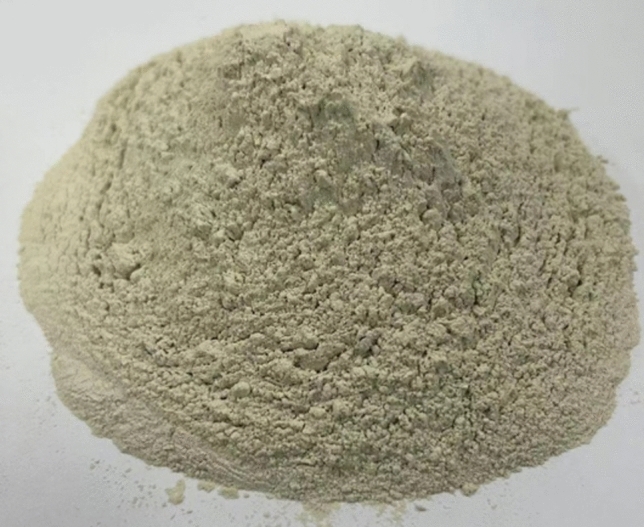
Bentonite sample.

Compared with calcium bentonite, sodium bentonite has better chemical stability, dispersion properties, gelling properties, higher water absorption and expansion times and is more suitable for use as an additive in backfill materials. A specimen was selected from the sodium bentonite produced by Gongyi Hengxin Materials Co., Ltd., in Henan Province. The sample is shown in Fig. 2. The soil was found to be a white, fine powder, and according to the GB/T 50123–2019 "Standard for Geotechnical Test Methods", its water content was measured to be 14.8%, its density was 2.64 g/cm^3^, and the main physical property indices are shown in Table 1.

**Table 1:** Indicators of the physical performance of bentonite.

Montmorillonite content (%)	Expansion capacity (mg/g)	Cellulose content (ml/15 g)	Water absorption (%)	Wet compressive strength (MPa)
75	35	100.2	295	1.35

The specimens were analysed by XRD, its main components were montmorillonite, cristobalite, quartz and albite. SEM–EDS was used to determine the elemental content of the sample. Bentonite is mainly composed of Si, Al, Fe, Ca and other elements, and the main elemental contents are shown in Table 2.

**Table 2:** Chemical element content of a bentonite sample.

Element	Si	Al	Fe	Ca	Mg	Na	S
Quantity (%)	62.44	21.98	6.09	5.39	2.50	1.50	0.10

### Experimental method

The bentonite ore was crushed, and the samples were heat treated in a muffle furnace. According to the results of the TG-DSC, the DSC curves showed two obvious endothermic peaks near 100.76 ℃ and 685.31 ℃, and two flat endothermic peaks near 898.91 ℃ and 1128.56 ℃. Therefore, to facilitate the subsequent experimental work, the temperatures were rounded to 100 ℃, 700 ℃, 900 ℃, 1100 ℃, which were selected as the heat treatment temperatures. The samples were held at the set temperature for 1 h and cooled in the furnace, after which the heat treated samples were put into self-sealing bags for storage.

TG-DSC: The analysis was carried out using an STA 449 F3 Jupiter synchronous thermal analyser from Netzsch. The temperature range was selected to be 30–1300 ℃ and heating rate was 20℃/min^21,22^, the test gas atmosphere was selected to be nitrogen due to its more stable chemical properties.

XRD: The crystal structures of the bentonite sample and heat treated sample were characterized by means of the powder compacting method; a Shimadzu XRD-6100 X-ray diffractometer with copper as the target material was used, with continuous scanning, a scanning speed of 10°/min, a sampling spacing of 0.02°, a scanning range of 5 ~ 80°, a tube voltage of 40 kV, and a tube current of 30 mA. Quantitative analysis of XRD results using the X'Pert HighScore Plus (v. 5.2, PANalytical B.V. Almelo, The Netherlands, https://www.malvernpanalytical.com/en/products/category/software/x-ray-diffraction-software/highscore).

SEM‒EDS: The morphology of bentonite sample and its heat treatment products was observed using a Phenom ProX scanning electron microscope from Thermo Fisher Scientific with an operating voltage of 15 kV.

## Results and discussion

### Energy changes of bentonite during the heat treatment reaction process

**Figure 3:**
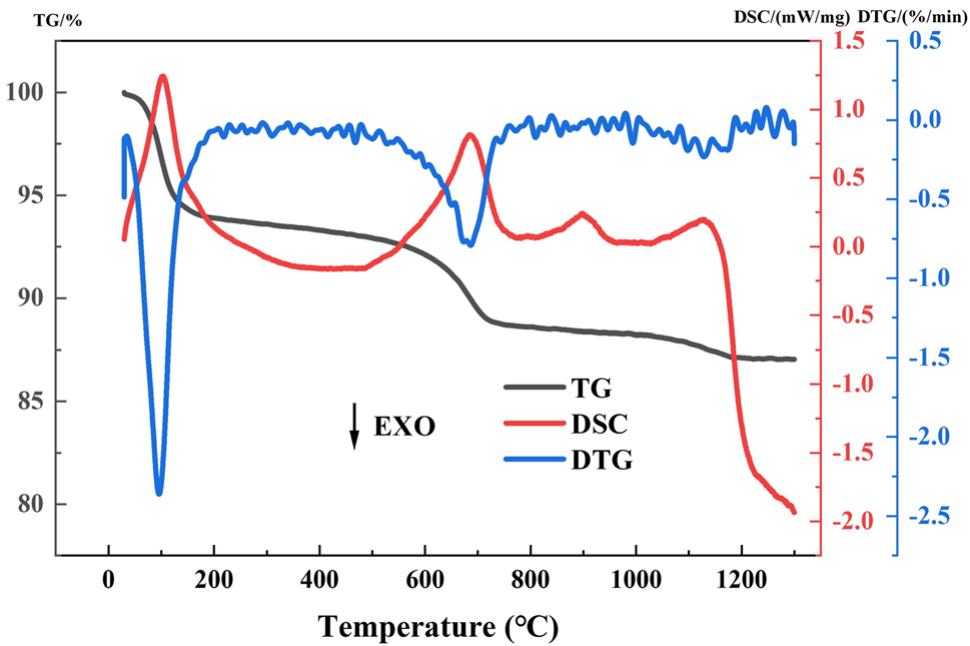
TG-DSC curves of bentonite.

The TG-DSC curves was shown in Fig. 3, TG and DTG curves showed that there were two obvious weight loss phases around 100 ℃ and 700 ℃, and the bentonite reached its maximum weight loss rates at 95.81 ℃ and 686.21 ℃, respectively. The DSC curves showed steep endothermic peaks near 100.76 ℃ and 685.31 ℃, and flat endothermic peaks near 898.91 ℃ and 1128.56 ℃. Based on the temperatures corresponding to the four endothermic peaks, the whole reaction process was divided into 4 phases: 30–400 ℃, 400–800 ℃, 800–1000 ℃, and 1000–1300 ℃.

Phase I (30–400 ℃): The absorption water and interlayer water on the surface of montmorillonite escaped with increasing temperature^23^, which led to the generation of the endothermic peak at 100.76 ℃ and the significant loss of thermogravimetry. The amount of thermogravimetric loss at this phase was 6.70%, reached the maximum loss rate of 2.36%/min at 95.81 ℃. The large amount of thermogravimetry loss and the high loss rate indicate that the absorption water and interlayer water content of montmorillonite was high and escaped rapidly.

Phase II (400–800 ℃): The constitution water of montmorillonite escaped with increasing temperature, and the two hydroxyl groups in the crystal structure were removed in the form of one water molecule, led to the generation of an endothermic peak and a significant loss of thermogravimetry at 685.31 ℃. The thermogravimetric loss in this phase was 4.70%, reached the maximum loss rate of 0.79%/min at 686.21 ℃. Compared with the first phase, the thermogravimetric loss and loss rate in this phase have decreased. The temperature at which montmorillonite undergoes dehydroxylation was an indicator for evaluating the heat resistance of montmorillonite and can reflect the thermal stability of bentonite^24^. Although the constitution water of montmorillonite is removed at this phase and its original characteristics begin to be lost, the montmorillonite crystal still maintained its original structure, with only layered structure distortion and torsion, and no obvious amorphization.

Phase III (800–1000 ℃): The crystal structure of montmorillonite began to break down, disintegrate and produce amorphous phase^25^, which led to an endothermic peak at 898.91 ℃, and the original layered structure of montmorillonite disappeared to produce anhydrous montmorillonite. The amount of thermogravimetric loss at this phase is 0.38%, and the thermogravimetry remains basically unchanged.

Phase IV (1000–1300 ℃): Quartz was transformed into cristobalite by high-temperature heat treatment^26^, and quartz absorbed heat during the transformation process resulting in an endothermic peak at 1128.56 ℃. The exothermic process that followed resulted from the recrystallization of the amorphous phase of bentonite. The melting point of albite is typically around 1100 °C^27^, and thus, the observed endothermic peak is attributed to the phase transition of quartz and the melting of albite. The subsequent exothermic process is due to the recrystallization of the amorphous phase of bentonite.

### Variation in the XRD patterns of bentonite heat treated at different temperatures

**Figure 4:**
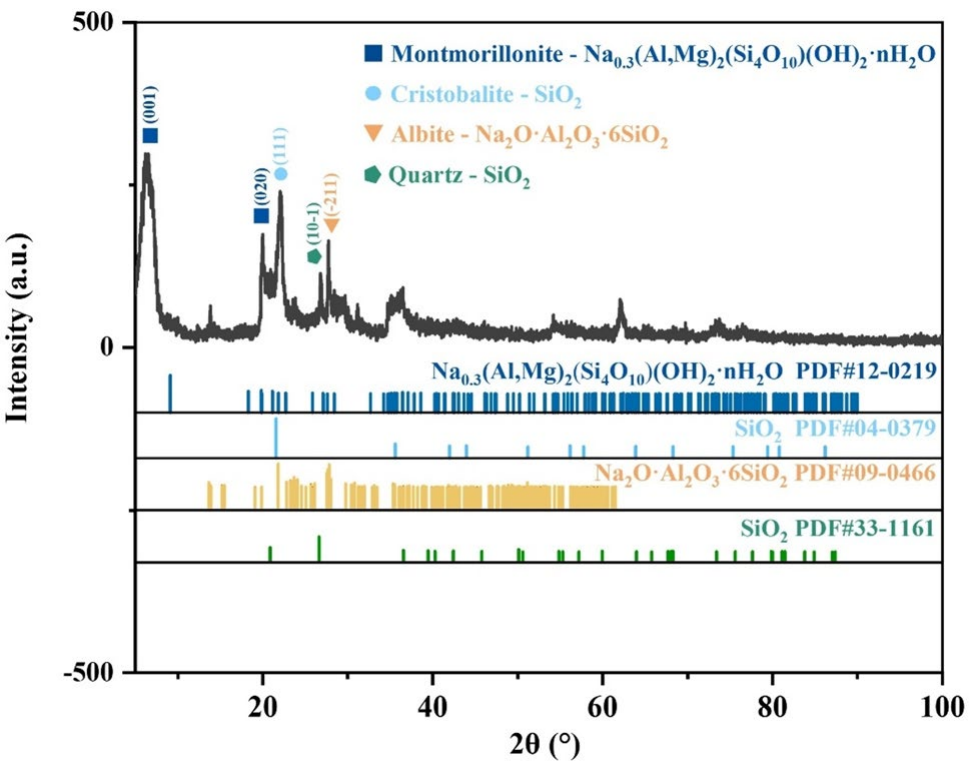
XRD pattern of raw bentonite ore.

**Figure 5:**
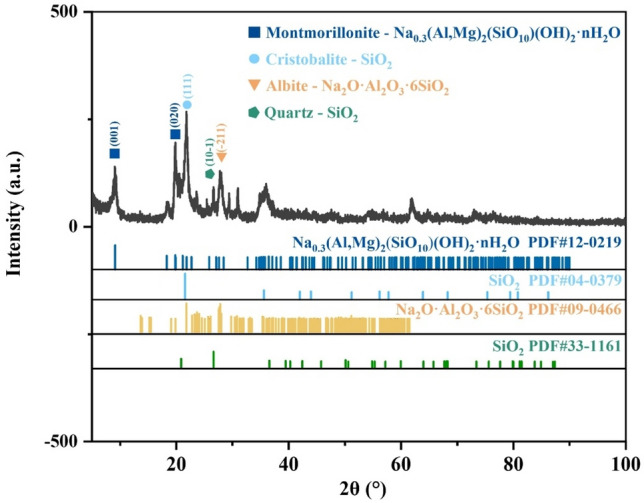
XRD pattern of bentonite after heat treatment at 100 ℃.

**Figure 6:**
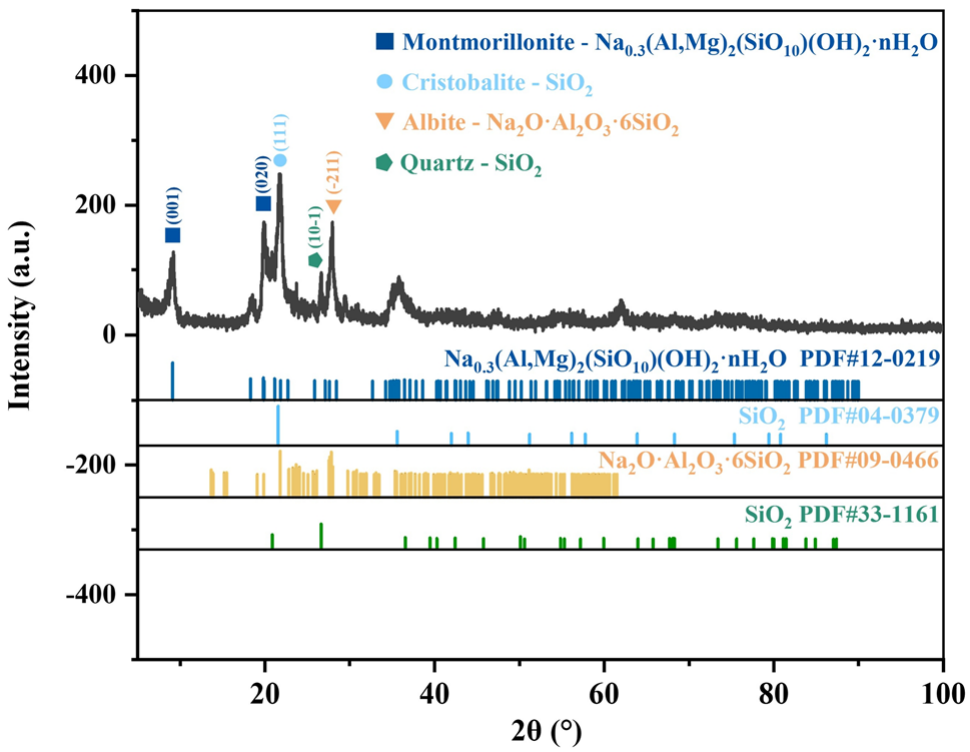
XRD pattern of bentonite after heat treatment at 700 ℃.

**Figure 7:**
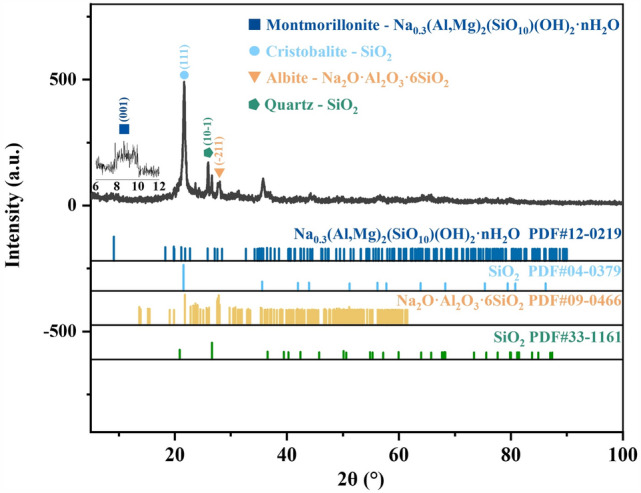
XRD pattern of bentonite after heat treatment at 900 ℃.

**Figure 8:**
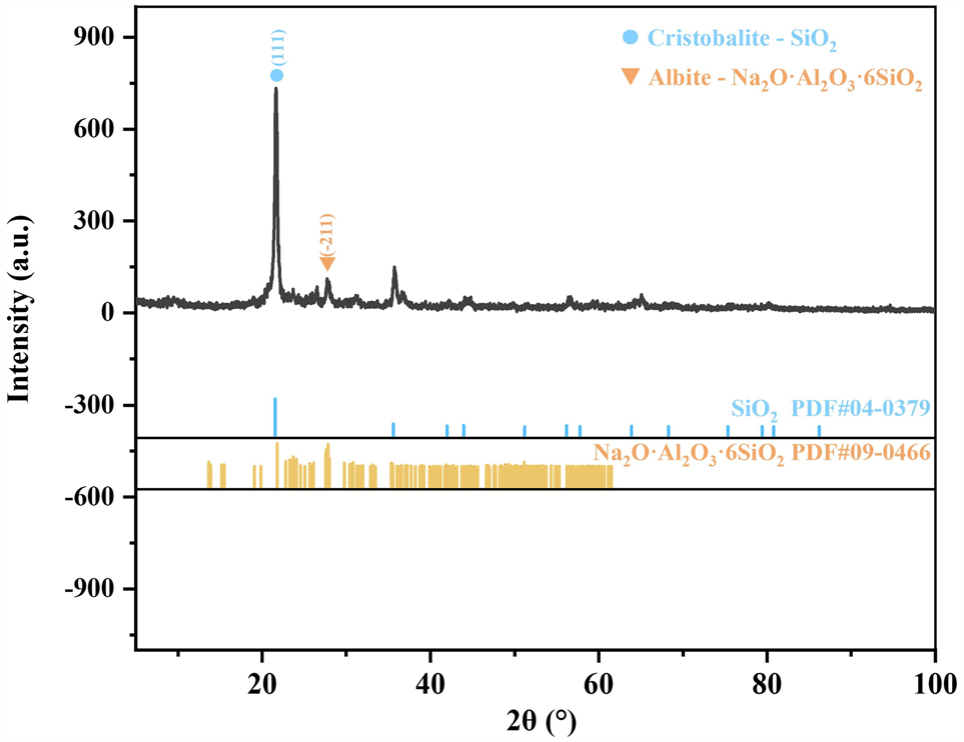
XRD pattern of bentonite after heat treatment at 1100 ℃.

To investigate the phase changes of bentonite during heat treatment, XRD analysis was conducted on the raw bentonite and its thermally treated products. Figures 4, 5, 6, 7, 8 display the XRD patterns of samples subjected to various heat treatment temperatures. The analysis reveals that the primary phases in the raw bentonite include montmorillonite, cristobalite, albite, and quartz. The diffraction peak for the (001) plane of montmorillonite in the raw bentonite appears at 2θ = 6.52° with a peak height of 298 and an interlayer spacing of 14.747 Å. After treatment at 100 °C, the diffraction peak shifts to 2θ = 9.08° with a reduced peak height of 140 and an interlayer spacing of 10.021 Å. This reduction in peak height by 158 and interlayer spacing by 4.726 Å indicates that the loss of adsorbed and interlayer water leads to the disappearance of the hydration layer, resulting in a decreased interlayer spacing and lower diffraction peak intensity^28^. Following treatment at 700 °C, the diffraction peak for the (001) plane of montmorillonite appears at 2θ = 9.2° with a peak height of 128, further reduced due to the loss of constitution water. After treatment at 900 °C, the montmorillonite diffraction peak significantly diminishes, and a broad hump appears near 2θ = 22°, suggesting the decomposition of montmorillonite into an amorphous phase at this temperature^29^. Following treatment at 1100 °C, the diffraction peaks for montmorillonite and quartz disappear, while a diffraction peak for the (111) plane of cristobalite emerges at 2θ = 21.64° with a peak height of 734, an increase of 242 compared to the cristobalite peak at 900 °C.

**Figure 9:**
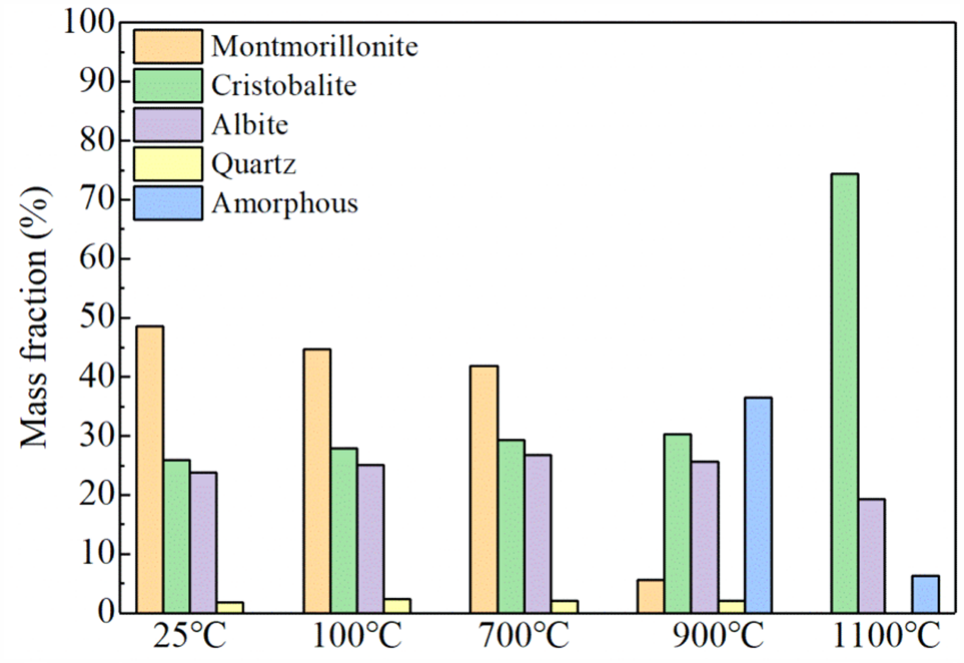
Phase contents of bentonite samples under different heat treatment conditions.

Figure 9 illustrates the phase composition of bentonite samples under different heat treatment conditions, calculated using the Rietveld whole pattern fitting method. In the raw bentonite, montmorillonite constitutes 48.49%, cristobalite 25.94%, albite 23.74%, and quartz 1.83%.

After heat treatment at 100 °C, the montmorillonite content decreases to 44.79%, cristobalite increases to 27.87%, albite to 25.03%, and quartz to 2.31%. These changes in phase proportions are attributed to the loss of water, which affects the total mass but does not alter the actual quantity of each phase. At 700 °C, the montmorillonite content further declines to 41.86%, while cristobalite rises to 29.28%, albite to 26.71%, and quartz slightly drops to 2.15%. The changes observed at this temperature are similar to those at 100°C, primarily driven by dehydration.

Upon heating to 900 °C, the content of cristobalite, albite, and quartz remains relatively unchanged; however, montmorillonite reduces drastically to 5.54%, and an amorphous phase emerges, constituting approximately 36.56% of the sample. DSC analysis indicates that the decomposition of montmorillonite occurs between 800 °C and 1000 °C, corresponding to the third endothermic peak on the DSC curve. The entire decomposition process begins at 800 °C and concludes at 1000 °C, with the maximum decomposition rate occurring at 900 °C. Residual montmorillonite is present because the heat treatment temperature was not sufficient to complete the amorphization process.

At 1100 °C, montmorillonite and quartz peaks disappear, and the content of cristobalite increases to 74.36%, while albite decreases to 19.31%, and the amorphous phase is reduced to 6.33%. The increase in cristobalite content is due to the recrystallization of the amorphous phase. According to Zhai et al.^30^, the recrystallization of amorphous bentonite leads to the formation of magnesium aluminum silicate (MgAl_2_Si_4_O_12_), which is a metastable phase that further decomposes into cristobalite. The disappearance of quartz is also a result of its conversion to cristobalite under high-temperature treatment. The reduction in albite content is explained by its melting point being around 1100 °C, at which point it begins to melt and form an amorphous liquid phase, accounting for the persistence of the amorphous phase even after recrystallization.

According to the research by Rehman et al.^31^, the heat treatment of clay minerals transforms their crystalline structure into an amorphous phase, significantly enhancing their pozzolanic activity. However, sodium-based bentonite exhibits a different behavior: the amorphous phase content initially increases with rising temperature but subsequently undergoes recrystallization with continued heating, which reduces the pozzolanic activity of the thermally treated clay^32^. Therefore, to achieve both enhanced compressive strength and sufficient expansiveness in the material, it is crucial to maintain the presence of both montmorillonite and the amorphous phase in bentonite. Based on the previously mentioned TG-DSC and XRD analyses, amorphization begins at 800 °C and is nearly complete around 900 °C, leaving 5.54% of the montmorillonite intact. Consequently, the optimal heat treatment temperature range is 800–900 °C.

### Changes in the microscopic morphology of bentonite heat treated at different temperatures

**Figure 10:**
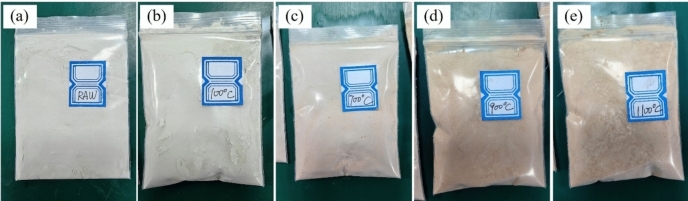
Colour change of bentonite: (**a**) raw ore, (**b**) temperature 100 ℃ (**c**) temperature 700℃, (**d**) temperature 900℃, and (**e**) temperature 1100℃.

First of all, macroscopic observations revealed that the colour of the specimen gradually changed from white to red as the heat treated temperature increased, as shown in Fig. 10.

According to the basic theory of solid-phase sintering, for stacked powders at high temperatures, the surface free energy of the material decreases, and as a result of the mutual diffusion of substances, the original microscopic discrete particles form a continuous solid structure, with a concomitant increase in the strength of the sample^33^. Prior to heat treated bentonite was composed of many particles, which contained pores; as the heat treated temperature increased, the edges of the particles appeared to melt, changing the shapes of the particles and pores and changing the surface energy of the particles; Diffusion resulted in the interparticle filling of pores, which resulted in a decrease in the porosity and the appearance of grain boundaries between the particles. As the heat treated temperature further increased, the size of the grain boundaries gradually increased, causing the bentonite clay to become denser, and recrystallization occurred.

**Figure 11:**
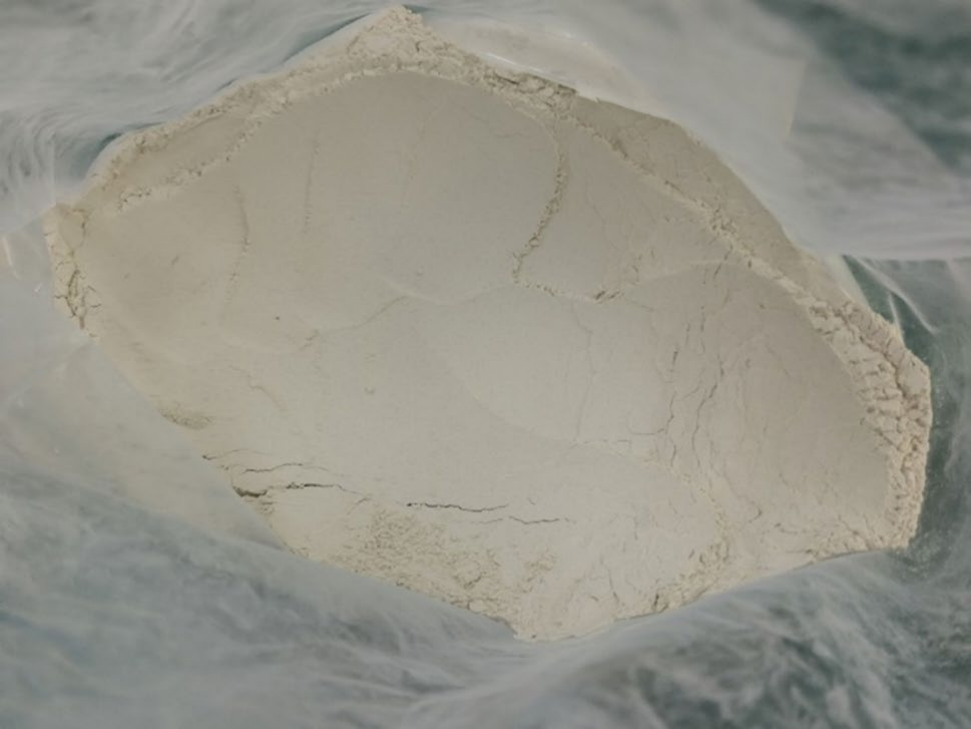
Macroscopic photographs of raw bentonite ore

**Figure 12:**
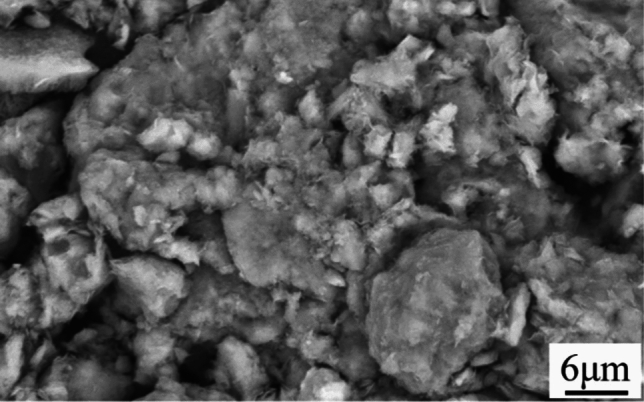
SEM image of the raw bentonite ore magnified 10000 times.

**Figure 13:**
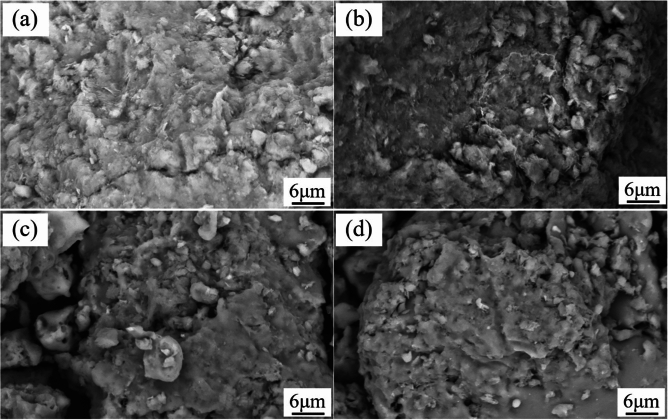
SEM images of bentonite heat treated products at a magnification of 10000 times: (**a**) temperature 100℃, (**b**) temperature 700℃, (**c**) temperature 900℃, and (**d**) temperature 1100℃.

**Figure 14:**
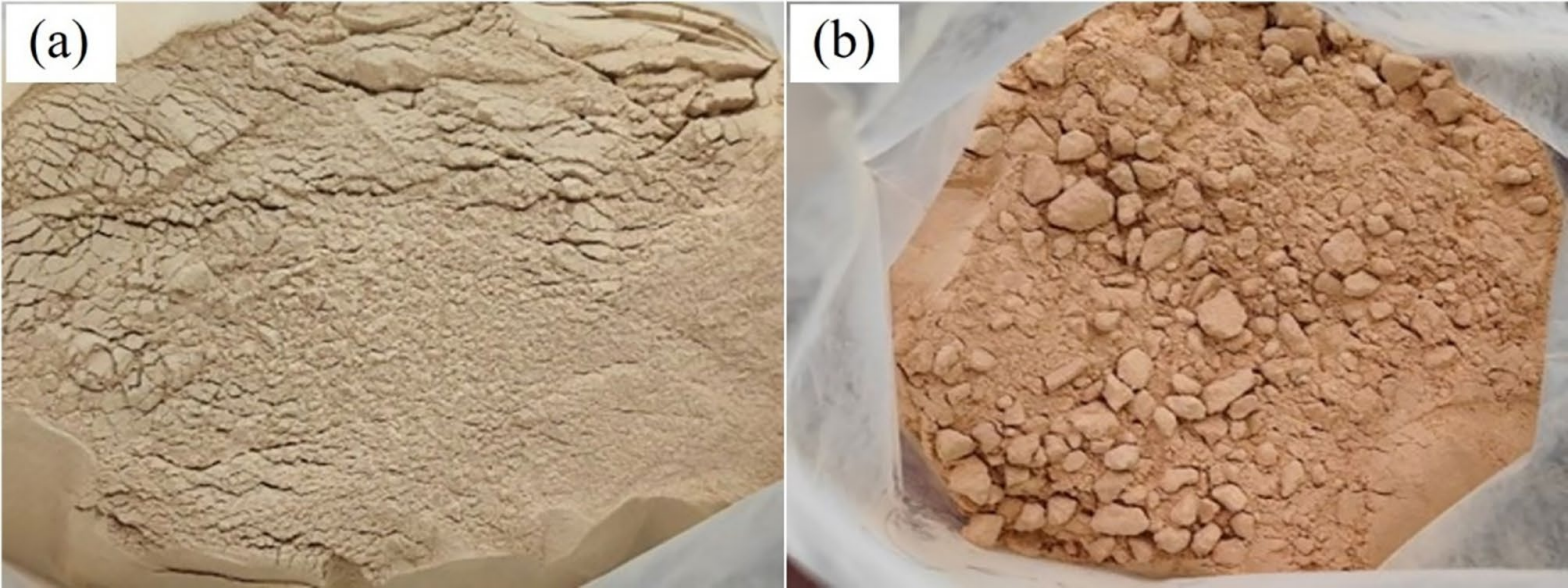
Macroscopic photographs of bentonite heat treated products: (**a**) temperature 700 ℃, (**b**) temperature 900 ℃.

At room temperature, the bentonite raw ore is white, no obvious agglomeration phenomenon, as shown in Fig. 11, and the SEM image in Fig. 12 shows that the bentonite raw ore particles were uneven in size and had irregular layered aggregates with rough surfaces and no obvious curling at the edges. Bentonite after heat treatment at 100 ℃, SEM image was shown in Fig. 13a, which no significant change from the raw ore; after heat treatment at 700 ℃, SEM image was shown in Fig. 13b, which show that the edges of the bentonite particles were curled because of the constitution water escaped, at the macroscopic level, the colour of bentonite changed, and the soil remained powdery, as shown in Fig. 14a; after heat treatment at 900 ℃, SEM image was shown in Fig. 13c, the bentonite particles became rounded, the edges appeared to be fused, the curls were reduced, and sticking occurred because of the structure of montmorillonite, which was originally lamellar, was disrupted, which, at the macroscopic level, was manifested as agglomeration of bentonite, as shown in Fig. 14b; after heat treatment at 1100℃, SEM image was shown in Fig. 13d, the bentonite particles further melted, most of the bentonite was in a molten state and adhered together, and the surface density increased, indicating the occurrence of recrystallization.

## Conclusions

Using TG-DSC, XRD, and SEM–EDS, this study investigates the effects of different heat treatments on raw bentonite. The goal is to determine the optimal heat treatment temperature range that ensures a synergistic effect between the expansiveness and pozzolanic activity of bentonite, thus broadening its application in filling materials. The specific conclusions are as follows:

- (1) The primary component of bentonite is montmorillonite. Montmorillonite loses adsorbed and interlayer water between 30 °C and 400 °C, undergoes dehydroxylation and loses constitution water between 400 °C and 800 °C, begins to decompose and forms an amorphous phase between 800 °C and 1000 °C, and undergoes recrystallization of the amorphous phase between 1000 °C and 1300 °C.
- (2) During the heat treatment process, the montmorillonite structure in bentonite is gradually destroyed and transformed into an amorphous phase. After treatment at 900 °C, the edges of the bentonite particles begin to melt and become smooth, showing signs of adhesion. Following treatment at 1100 °C, most of the bentonite is in a molten state, with increased surface density and significant recrystallization phenomena.
- (3) The optimal heat treatment temperature range is 800–900 °C. Within this temperature range, both montmorillonite and the amorphous phase coexist, which enhances the compressive strength of the material while maintaining its expansiveness.

## Data Availability

The datasets used and/or analysed during the current study are available from the corresponding author upon reasonable request.
